# Supplementary material for: A web-database of mammalian morphology and a reanalysis of placental phylogeny
Source: BMC Evol Biol. 2007 Jul 3;7:108. doi: 10.1186/1471-2148-7-108 (PMC1941728; doi:10.1186/1471-2148-7-108)
Supplement: Additional File 1 — Website data: The 196 characters first described in [17], along with the supplementary data indicated below, have been joined into a number of HTML files, all of which are linked to the "morphsite_bmc07.html" index page. The embedded links to the supporting files are functional, independent of PC or platform, provided that the directory structure remains intact (i.e., all files remain in a single folder, no subdirectories). Nexus files: Datafiles containing the morphological, indel, and aligned DNA sequence data, linked from the "morphsite_bmc07.html" index page. Table 1. Changes to the multiple sequence alignment of [9]. Table 2. Qualitative character-state corrections to the morphology matrix of [17]. Table 3. Summary of discrepancies between Appendices 1 and 2 of [17]. [file 1471-2148-7-108-S1.zip › asher-BMC07-website/char46.htm]

char46


character 46:
Dorsum sellae (Novacek, 1986, char. 37)
